# Multi-objective optimization and prediction of surface roughness and printing time in FFF printed ABS polymer

**DOI:** 10.1038/s41598-022-20782-8

**Published:** 2022-10-07

**Authors:** Arivazhagan Selvam, Suresh Mayilswamy, Ruban Whenish, K. Naresh, Vigneshwaran Shanmugam, Oisik Das

**Affiliations:** 1 Department of Mechanical Engineering, KPR Institute of Engineering and Technology, Coimbatore, Tamil Nadu India; 2 Department of Robotics and Automation Engineering, PSG College of Technology, Coimbatore, Tamil Nadu India; 3 Centre for Bio Materials, Cellular and Molecular Theranostics, Vellore Institute of Technology, Vellore, Tamil Nadu India; 4 Department of Chemical Engineering and Materials Science, University of Southern California, Los Angeles, 90089, USA; 5 Department of Civil, Environmental and Natural Resources Engineering, Luleå University of Technology, 97187 Luleå, Sweden; 6 Department of Mechanical Engineering, Saveetha School of Engineering, Saveetha Institute of Medical and Technical Sciences, Chennai, India

**Keywords:** Mechanical engineering, Polymers

## Abstract

In this study, fused filament fabrication (FFF) printing parameters were optimized to improve the surface quality and reduce the printing time of Acrylonitrile Butadiene Styrene (ABS) polymer using the Analysis of Variance (ANOVA), it is a statistical analysis tool. A multi-objective optimization technique was employed to predict the optimum process parameter values using particle swarm optimization (PSO) and response surface methodology (RSM) techniques. Printing time and surface roughness were analyzed as a function of layer thickness, printing speed and nozzle temperature. A central composite design was preferred by employing the RSM method, and experiments were carried out as per the design of experiments (DoE). To understand the relationship between the identified input parameters and the output responses, several mathematical models were developed. After validating the accuracy of the developed regression model, these models were then coupled with PSO and RSM to predict the optimum parameter values. Moreover, the weighted aggregated sum product assessment (WASPAS) ranking method was employed to compare the RSM and PSO to identify the best optimization technique. WASPAS ranking method shows PSO has finer optimal values [printing speed of 125.6 mm/sec, nozzle temperature of 221 °C and layer thickness of 0.29 mm] than the RSM method. The optimum values were compared with the experimental results. Predicted parameter values through the PSO method showed high surface quality for the type of the surfaces, i.e., the surface roughness value of flat upper and down surfaces is approximately 3.92 µm, and this value for the other surfaces is lower, which is approximately 1.78 µm, at a minimum printing time of 24 min.

## Introduction

Digital manufacturing gained attraction among manufacturing industries due to its high accuracy, adapting the conditions, design freedom and shortened lead time. Additive Manufacturing (AM) is one of such technologies which are expanding its versatility into many present-day applications such as aviation, construction, healthcare, food, etc.,^1^. A three-dimensional CAD (Computer-Aided Design) file of given geometry including complex shapes can be built layer by a layered approach with designated process parameters^2,3^. By incorporating AM techniques over traditional manufacturing, it is possible to reduce material waste, reduce design to manufacturing time, and increase design flexibility. AM techniques can build complex geometries with multi-material in a single processing phase^4–6^. Fused Filament Fabrication (FFF) is the well-known extrusion-based AM technique that is widely considered for fabricating polymers for prototyping and functional requirements^7–9^. FFF provides the feasibility of printing complex 3D architecture with low cost and limited material wastage.

**Figure 1:**
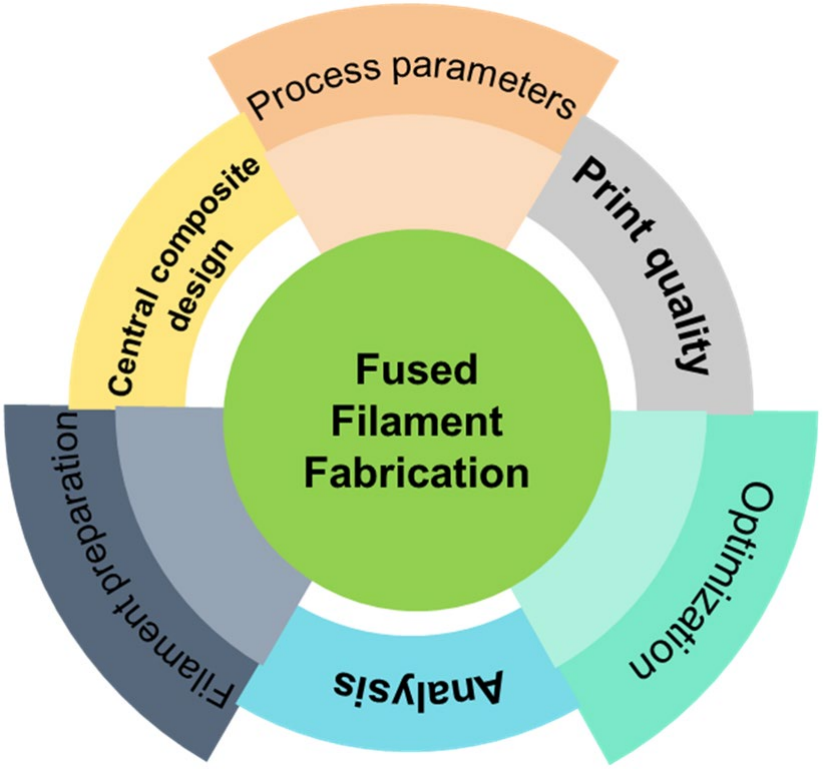
FFF materials-process-part performance.

Numerous researchers worked on the development of processes and materials in FFF, with a focus on observing the relationship between FFF process parameters and the printing material, which is related to the mechanical properties of the final part. The impact of various such parameters (either individually or combined) on printing material is expressed in mechanical integrity, printing performance, functional behavior, and surface characteristics^7,10–15^. For instance, it was observed that the infill density and layer thickness have significantly influenced the tensile strength of ABS polymer^16^. High infill density gives high tensile strength and good surface characteristics for PETG material^17^. Apart from changes in internal process parameters, some external changes were also considered to obtain the expected mechanical performance of printed material by adding carbon fibers (short/lengthy) along with polymers^18^. These compositions may not consistently spread throughout the polymer structure which yields non-uniform mechanical performance of printed parts. So, it is necessary to identify the suitable process parameters for the defined material with the optimal condition to secure the quality 3D printed parts^19^. The overview of the interaction between materials, process, and part characteristics in the FFF process is illustrated in Fig. 1.

The identification of optimal parameters can be possible by applying optimization techniques. Taguchi is one of the conventional optimization techniques used to find out optimal process parameters^20,21^. Afterward, many optimization techniques were used by researchers to obtain the precise parameters which directly improvise the mechanical performance of FFF processed polymers. Response surface methodology (RSM), Genetic Algorithm (GA) and Particle Swarm Optimization (PSO) interacted between different polymer materials and process parameters as an outcome of the mechanical performance of FFF fabricated parts^22–24^. Multi-objective optimization brings out even finer optimal values to enhance the mechanical performance of extruded 3D parts^25^. Grey Relational Analysis (GRA) is a beneficial tool to analyze the correlation between sequences with minimal data where experiments are time-consuming and expensive^26^. The compressive strength and hardness of zirconia reinforced alumina using GRA reduced the number of trials which helped to save time, cost and material wastage and produced accurate optimal values^27^. In another investigation, GRA coupled with RSM to optimize FFF process parameters with a multi-response function which yields precise values of each parameter and their significance. Apart from that the experimental runs also decreased which reduced the printing time, material usage and cost incurrence^28^. The function of GRA is to achieve multiple performance characteristics of machined or 3D printed parts with fewer experimental runs and considerable economic benefits^29^. To maximize the tensile strength and hardness value of the LM24 Al alloy, the multi-objective function was developed by the weighted sum method. The developed mathematical model has used a fitness function for GA which predicts a maximum tensile strength of 272.2 (N/mm^2^) and hardness of 98.01 (BHN)^30^. The efficiency of the PSO algorithm was studied by convergence time and the accuracy of the predicted results. Compared to the results predicted by fuzzy set theory, GA and goal programming, the optimum result predicted by PSO was better^31^. The optimum parameters of the squeeze casting process were predicted by GA, PSO and crowing distance-based multi-objective PSO are compared to find the best optimization algorithm. The PSO algorithm performs better than GA to predict the best combination of parameters in minimum time^32^. GA is employed to solve multi-objective functions developed based on the response weights. The combination of process parameters to minimize surface roughness and maximize YS, UTS and hardness of the squeeze casting process was effectively predicted by GA^33^. The WASPAS method is a combination of WSM and WPM to identify the best alternatives based on ranking order. The impact of WPM and WSM on ranking order is controlled by the varying parameter λ value from (0 to 1)^34^. Multi-Criteria Decision Making (MCDM) methods are one of the popularly used tools to solve multi-objective problems. The optimum parameters to minimize surface roughness, dimensional deviation and material removal rate of the end milling process was effectively predicted through MCDM tools such as WASPAS (Weighted Aggregated Sum Method), AHP and VIKOR approach^35^. To confirm the effectiveness and applicability of the WASPAS method, the best combination of variable parameters to attain the output responses of five non-traditional machining processes is identified through the WASPAS rank order. WASPAS solve both single and multi-objective problem effectively, moreover, the effectiveness and the accuracy of the decision-making process is varied by giving optimum λ value^36^. The best combination parameter for the Abrasive Jet Machining process was obtained through the WASPAS method. The result showed that the WASPAS method can solve conflict multi-objective problems^37^. The top six Evolution Algorithms such as GA, PSO, BBO, WCA, BA and SMS are evaluated by the WASPAS method to solve the multi-objective function of the Karun4 reservoir. The WASPAS raking order PSO (1), WCA (2), GA (3), BBO (4), BA (5) and SMS (6) illustrates that compared to other EAs PSO secure 1st rank and performance well in solving multi objection function^38^.

The literature survey conveys that process parameters such as printing speed, layer thickness and nozzle temperature are highly influencing the surface quality and printing time of FFF printed components. There are limited studies carried about on overall surface finish characteristics with respect to the least printing time. Hence, there is a need to find the optimum printing parameters to fabricate high-quality components at minimum printing time. There are several studies available related to the experimental characterization of FFF printed ABS polymer^39–42^. However, the studies related to multi-objective optimization techniques and WASPAS ranking for predicting and optimizing the FFF process printing parameters of polymers using statistical analysis tools are limited. These studies are essential to minimize the number of experiments and reduce the printing time. ABS and PLA are being widely used polymers in the FFF technique and ABS is harder to print compared with PLA. For mass production, the time consumption for obtaining the product should be minimized without scarifying part quality. By considering these aspects, the present research was carried out to find multiple performances such as overall surface finish and printing time of FFF processed ABS polymer, which can be used for various product development and optimization applications.

## Materials and methods

In this study, ABS polymer was used. The most used materials in the FFF technique are ABS and poly lactic acid (PLA) due to their flexibility in manufacturing, availability, and low cost. Compared to PLA material, fabricating a component with ABS material is challenging. The operating temperature for ABS (220–245 °C) material is higher when compared with PLA (180–210 °C). The selection of parameters for this study correlated with minimum printing time with enhanced surface quality. Printing speed, layer thickness and nozzle temperature are the important parameters in influencing over required criteria of meeting minimum printing time with higher quality printed parts^43^. Hence, there is a need to optimize the input parameters, to enhance the surface quality of parts fabricated with ABS material at minimum time. The mechanical properties of ABS are given in Table 1.

**Table 1:** Mechanical Properties of ABS.

Properties	Unit	Value
Tensile strength	(MPa)	29.8–43
Young modulus	(GPa)	1.79–3.2
Elongation at break	(%)	10–15
Flexural modulus	(GPa)	1.6–2.4

### Specimen fabrication

In this research, all the specimens were fabricated using a low-cost FFF Ender3 3D printer with dimensions of 4 cm x 4 cm x 1.5 cm. To measure the surface roughness and printing time, the developed 3D model was converted from .IGES format to .STL (STereoLithogrphy) format. To fabricate specimens, the imported .STL file format was converted to G-code and fed into the 3D printer through an SD card port. Specimens were printed by varying the input process parameters, namely printing speed, layer height and extruder temperature. The Other parameters namely infill (20%), build orientation, build plate temperature (110 °C), shell thickness (1.6 mm), roof thickness (1.1 mm) and raster angle(+ 45°/− 45°) were maintained constant, these values were selected based on the initial experimentation.

### Specimen characterization

**Figure 2:**
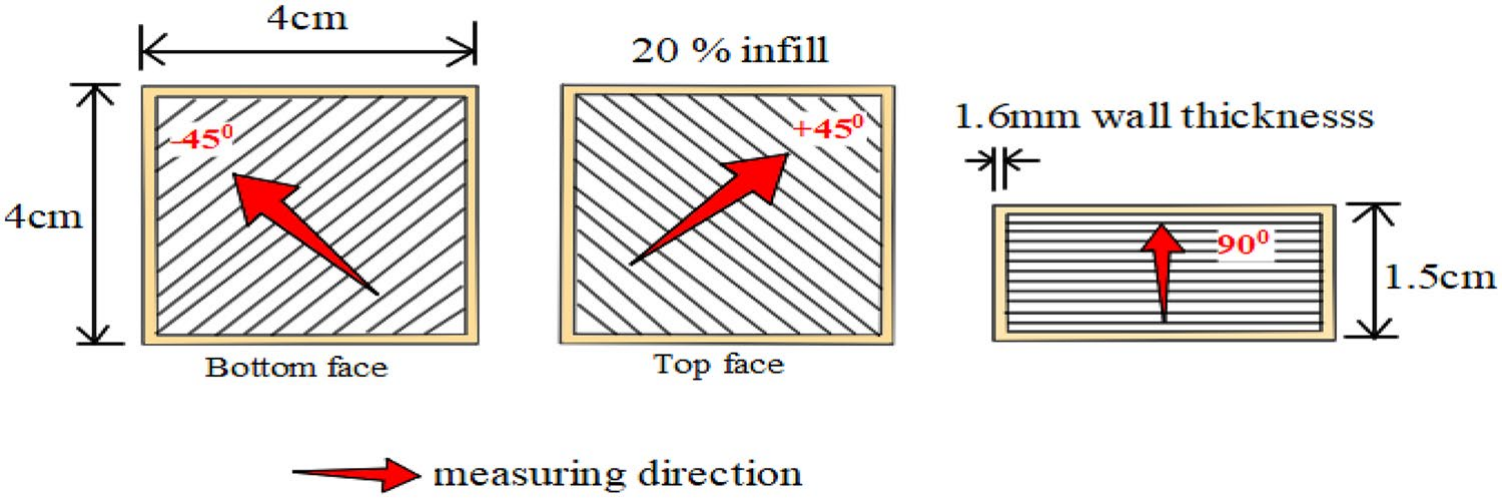
Specimen dimensions and measuring direction.

In general, the surface quality of the top and bottom surfaces of the FFF part is not equal, compared to the side surfaces. Therefore, in this research, the average of top and bottom surface roughness values (SR^*TB*^) and the average of four sides surface roughness value (SR^*S*^) of specimen were measured using the surface roughness measuring instrument Surftronic (contact type). The data related to the SR^*S*^ and SR^*TB*^ are provided in the supplementary files. The dimensions of the fabricated specimen and the measuring direction are shown in Fig. 2. The time taken for printing each specimen is measured using a stopwatch and the average surface roughness values are given in Table 3.

**Table 2:** FFF process parameters and levels.

FFF parameters	Units	Levels		
		− 1	0	+ 1
Printing speed	mm/s	60	105	150
Layer thickness	mm	0.1	0.2	0.3
Nozzle temperature	°C	220	232.5	245

### Design of experiment

**Figure 3:**
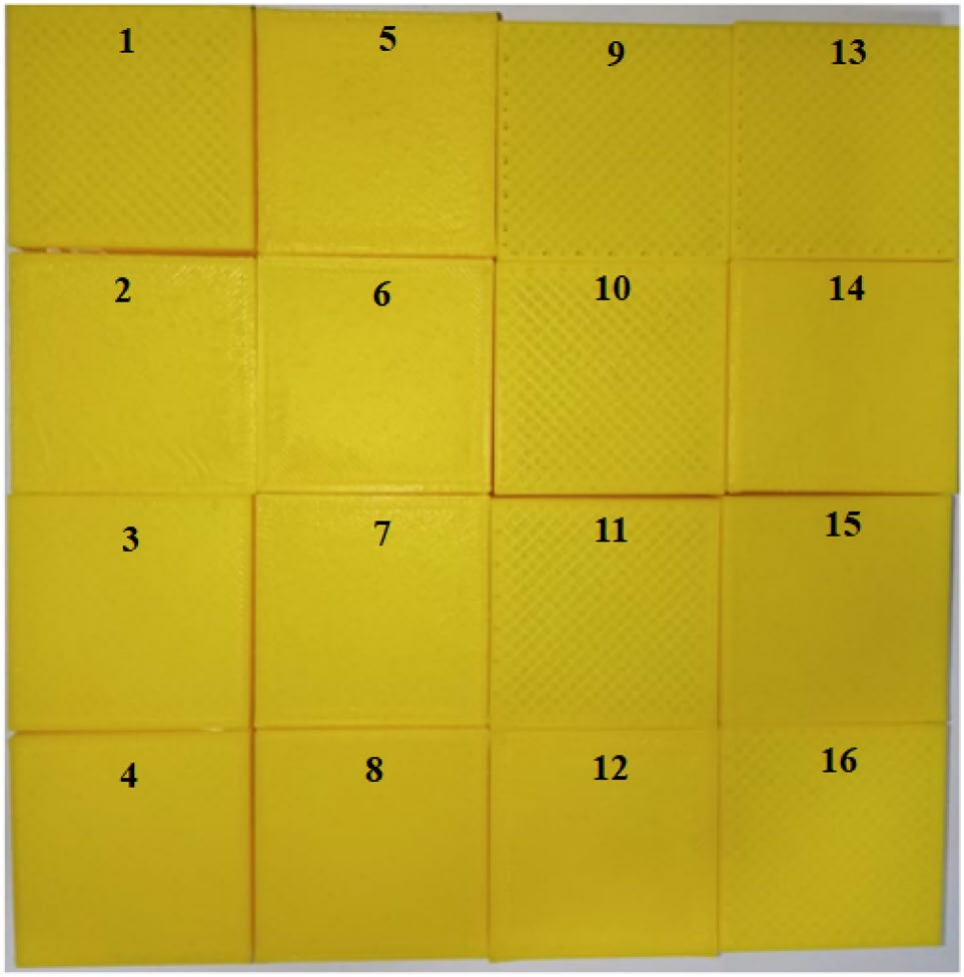
Fabricated FFF specimens.

To find out the correlation between the identified process parameters and the output response DoE table was constructed using JMP software. The significant process parameters identified from the literature survey such as printing speed (mm/s), layer thickness (mm) and nozzle temperature (°C) values are taken at 3 levels − 1, 0 and + 1 as shown in Table 2. The fabricated specimens as per DoE are shown in Fig. 3.

**Figure 4:**
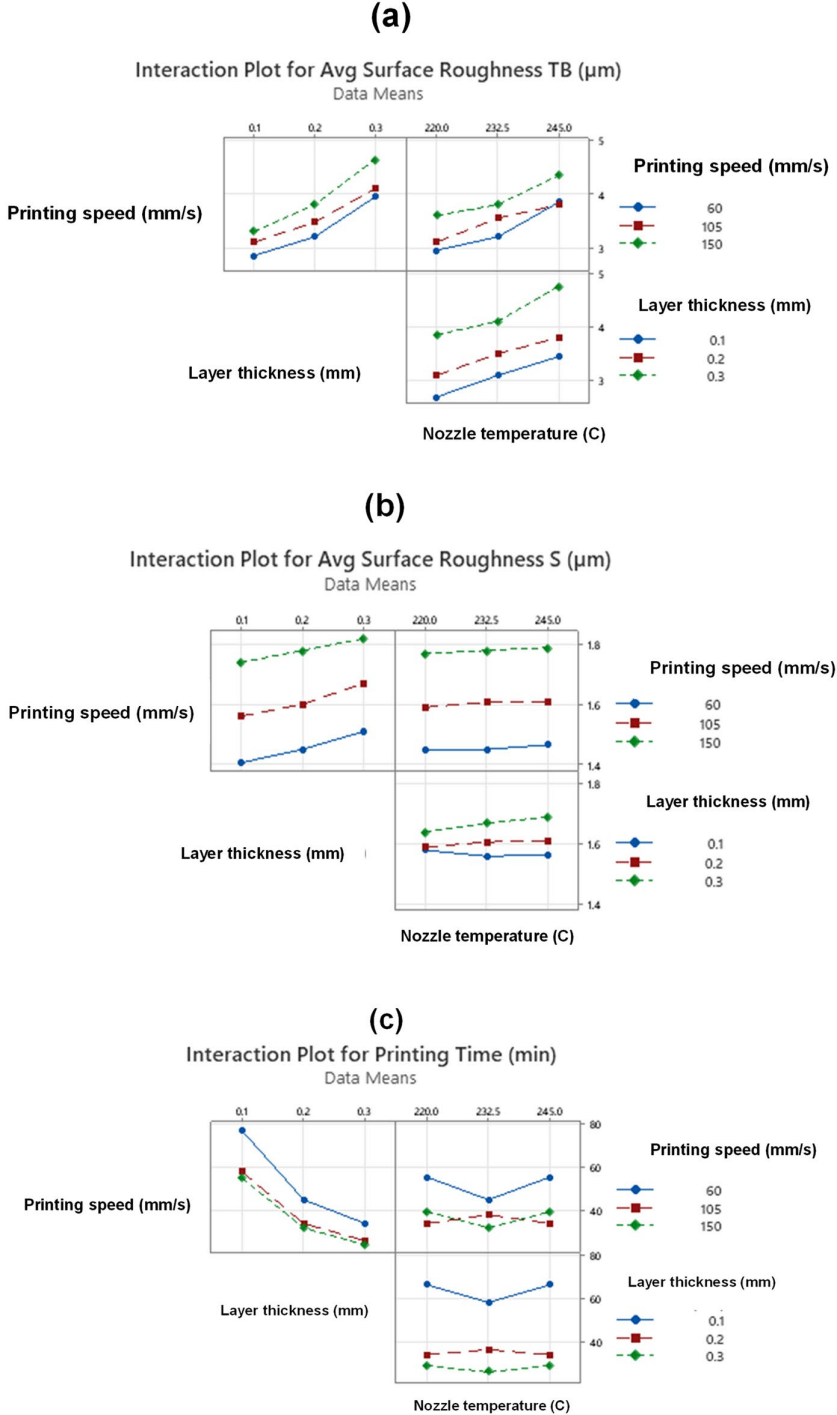
Interaction plot between FFF parameters versus part characteristics.

To minimize the number of experiments in Central Composite Design (8 factorial design + 6 axial point + 2 center points), 16 experiments were conducted and the measured responses, average Surface Roughness SR^*TB*^, average Surface Roughness SR^*S*^ and Printing Time (min) are depicted in Table 3. The interaction between FFF process parameters with part characteristics is depicted in the interaction plot (Fig. 4).

**Table 3:** Measured surface roughness SR^*TB*^, SR^*S*^ and printing time.

S. No	FFF parameters			Responses		
	Printing speed	Layer thickness	Nozzle temperature	Average surface roughness SR^*TB*^	Average surface roughness SR^*S*^	Printing time (PT)
	mm/s	mm	°C	Ra(µm)	Ra(µm)	(min)
1	105	0.2	232.5	3.5	1.6	34
2	150	0.1	220	2.9	1.75	55
3	60	0.2	232.5	3.2	1.45	45
4	60	0.3	220	3.4	1.49	34
5	105	0.3	232.5	4.1	1.67	26
6	60	0.3	245	4.5	1.53	34
7	105	0.2	245	3.8	1.61	34
8	60	0.1	245	3.2	1.4	77
9	105	0.2	220	3.1	1.59	34
10	105	0.1	232.5	3.1	1.56	58
11	105	0.2	232.5	3.5	1.6	34
12	150	0.3	220	4.3	1.79	24
13	150	0.2	232.5	3.8	1.78	32
14	60	0.1	220	2.5	1.41	77
15	150	0.1	245	3.7	1.73	55
16	150	0.3	245	5	1.85	24

Figure 4 shows how variables such as surface roughness and printing time depend on one another as seen in the interaction plots. Printing speed, layer thickness, and nozzle temperature all show a proportional increase with each other for flat upper and lower surfaces in Fig. 4a. A similar pattern was seen in the relationship between layer thickness and printing speed forside surface roughness. Printing speed vs. nozzle temperature and layer thickness vs. nozzle temperature both exhibits virtually constant values concurrently in Fig. 4b. The relationship between layer thickness and printing speed is shown in Fig. 4c. The printing time increases when the layer thickness is low, and the printing speed is low. The printing time is greatly decreased while printing at the maximum speed with the thickest layer. The effect of the nozzle temperature on printing time is minimal when compared to other factors.

## Results and discussion

### Mathematical model

To derive an effective regression model and to explore the influence of process parameters on the responses, MiniTab software was used. The mathematical model developed to predict the Surface Roughness (µm) and Printing Time (min) of fabricated FFF specimens using Eqs. (1), (2) and (3), respectively.

1 $${SR}^{TB}=k {h}^{p}{i}^{q}{j}^{r} \varepsilon$$

2 $${SR}^{s}=k {h}^{p}{i}^{q}{j}^{r} \varepsilon$$

3 $$PT=k {h}^{p}{i}^{q}{j}^{r} \varepsilon$$

where *SR*^*TB*^ is the average surface roughness (µm) measured on the top and bottom surface of the specimen, *SR*^*S*^ is the average surface roughness (µm) measured inside the face, *PT* is the printing Time in (min), h, i and j represent the printing speed (mm/s), layer height (mm), and nozzle temperature (°C), respectively. p, q and r represent the estimated parameters of the developed model. The experimental error is represented as ɛ.

From the measured responses of 16 specimens in Table 3, a mathematical model for SR^TB^, SR^S^ and PT represented in Eqs. (4), (5) and (6) was developed by estimating the FFF parameter coefficients. Where model parameters x_1_, x_2_ and x_3_ represent the printing speed, layer thickness and nozzle temperature, respectively.

The significance of the developed model for SR^TB^ and its parameters were examined by the ANOVA. The SR^TB^ model *p-value* is lower than 0.0001 that indicates that the developed SR^TB^ model and its parameters x_1_, x_2_ and x_3_ were more significant. The R^2^ value of the SR^TB^ model was 96% that indicates that the model is reliable.

4 $${SR}^{TB}=-5.6966+0.0064{x}_{1}+5.9{x}_{2}+0.032{x}_{3}$$

The R^2^ value of Eq. (4) ${SR}^{TB}$ is 96%.

The significance of the developed model for SR^S^ and its parameters were examined using the ANOVA tool. The SR^S^ model *p-value* is lower than 0.0001 which indicates that the developed SR^S^ model and its parameters x_1_, x_2_ and x_3_ were more significant. The R^2^ value of the SR^S^ model is 99% that indicates that the model is reliable.

5 $${SR}^{S}=0.9717+0.0036{x}_{1}+0.48{x}_{2}+0.00072{x}_{3}$$

The R^2^ value of Eq. (5) ${SR}^{S}$ is 99%.

The significance of the developed model for *PT* and its parameters were examined using the ANOVA tool. The *PT* model *p-value* was lower than 0.0001 that indicates that the developed *PT* model and its parameters x_1_ and x_2_ are more significant, whereas the *p-value* of x_3_ parameter was greater than 0.0001 that indicates that it is not an appropriate parameter to fabricate the component based on the printing time. The R^2^ value of model *PT* was 100% that indicates that the model is reliable.

6 $$PT=305-0.814{x}_{1}-587{x}_{2}-1.23{x}_{3}+0.002427{x}_{1}^{2}+841.4{x}_{2}^{2}+0.00265{x}_{3}^{2}+0.667{x}_{1}{x}_{2}$$

The R^2^ value of Eq. (6) $PT$ is 100%.

From the ANOVA table based on the R^2^ value, *P*-value and lack of fit, the effectiveness of the developed model was validated and used as a fitness function for optimization. To achieve a high surface quality finish at the lowest printing time, 50% weightage is given to Printing Time (*PT*), and 25% of weightage is given to Top and Bottom Surface Roughness (${SR}^{TB}$) and 25% weightage is given to Surface Roughness of the side face (${SR}^{S}$). The weightage has been assigned by following the Equal-weights method (50% for printing time & 50% for surface roughness) as the total number of responses is 2^40^.The regression model developed to predict surface roughness and printing time was coupled to create a single regression model using the weighted sum method. The multi-objective function (X), to minimize printing time and surface roughness value, is given in Eq. (7)

7 $${\text{Multi - objective function}}\;({\text{Minimization}}){\text{X}} = {\text{W}}_{1} (PT) + {\text{W}}_{2} (SR^{TB} ) + {\text{W}}_{3} (SR^{S} )$$

where W_1_ = 0.5,W_2_ = 0.25 and W_3_ = 0.25 represents the corresponding weights of responses *PT*, *SR*^*TB*^ and *SR*^*S*^.

8 $$\begin{gathered} {\text{X}} = 0.{5}\left( {305 - 0.814x_{1} - 587x_{2} - 1.23x_{3} + 0.002427x_{1}^{2} + 841.4x_{2}^{2} + 0.00265x_{3}^{2} + 0.667x_{1} x_{2} } \right) \hfill \\ + 0.25\left( { - 5.6966 + 0.0064x_{1} + 5.9x_{2} + 0.032x_{3} } \right) + 0.25\left( {0.9717 + 0.0036x_{1} + 0.48x_{2} + 0.00072x_{3} } \right) \hfill \\ \end{gathered}$$

9 $$X=149.95-0.4038{x}_{1}-290.55{x}_{2}-0.599{x}_{3}+0.0012135{x}_{1}^{2}+420.7{x}_{2}^{2}+0.001325{x}_{3}^{2}+0.333{x}_{1}{x}_{2}$$

The above Eq. (9) is used as a fitness function to perform PSO to predict the optimum parameters in enhancing the overall surface quality of 3D printed parts at minimum printing time.

### Particle swarm optimization

Dr. James Kennedy, Dr. Russel and Eberhart inspired by the birds foraging behavior developed an algorithm to predict optimum values for the given objective function. To improve the surface quality of FFF parts at minimum printing time PSO technique is employed.

10 $$({\text{Minimize}}){\text{X = W}}_{1} (PT) + {\text{W}}_{2} (SR^{TB} ) + {\text{W}}_{3} (SR^{S} )$$

The following steps are coded in MATLAB software in order to find the optimum solution for the multi-objective function given in Eq. (10).

Step 1: Program Start.

Step 2: Generate random values to initialize the particle.

Step 3: PSO parameters Setting Inertia Weight (w) = 0.5, Acceleration co-efficient (*C*_1_ = 1, *C*_2_ = 1) and maximum iteration (*ni*)) is set to 300 and population (*np*) is set to 100.

Step 4: Minimization Objective Function (X = W_1_ (*PT*) + W_2_ (${SR}^{TB}$) + W_3_ (${SR}^{S}$).

Step 5: Find the global best and the local best.

Step 6: Update new velocity $({v}_{i}\left(k+1\right)= {v}_{i}\left(k\right)+{C}_{1}({p}_{i}-{x}_{i}\left(k\right)+{C}_{2}(G-{x}_{i}\left(k\right) )$ and new position (${x}_{i}\left(k+1\right)={x}_{i}\left(k\right)+{v}_{i}(k+1)$).

Step 7: Identify Min(X) and its optimum parameters.

Step 8: Print Min(X) and its minimum parameters.

Step 9: Program Stop if Max iteration reached or Min(X) reached.

The significant parameters of the PSO algorithm are the number of a particle (*np*), number of iterations (*ni*) and inertia weight (*w*). The number of particles was set to 100 which were sufficient to minimize the objective function. On the other hand, the maximum iteration was set to 300, since the PSO algorithm converged in less than 300 iterations. In this research, the inertia weight (*w*) which controls the convergence speed was set to 0.5. The acceleration co-efficient *C1* that controls the local best position and *C2* that controls the global best position, were set to a value equal to 1.To predict the optimum process parameters, the range is given in Eqs. 11(a), (11b) and (11c).

11a $$60 \le {\text{x}}_{1} \ge 150$$

11b $$0.1 \le {\text{x}}_{2} \ge 0.3$$

11c $$220 \le {\text{x}}_{3} \ge 245$$

The PSO algorithm predicted minimum value for the objective function (10) is given in Eq. (12).

12 $$149.95-0.4038\left(125.86\right)-290.55\left(0.295\right)-0.599\left(225.7\right)+0.0012135{\left(125.86\right)}^{2}+420.7{\left(0.295\right)}^{2}+0.001325{\left(225.7\right)}^{2}+0.333\left(125.86\right)\left(0.295\right)=13.915$$

The PSO predicted top 5 optimum parameters given in Table 4 are printing speed (x_1_), layer thickness (x_2_) and nozzle temperature (x_3_) which were within the given parameter range.

**Table 4:** PSO Algorithm predicted results.

S. No	FFF parameters			Output responses		
	Printing speed (mm/s)	Layer thickness (mm)	Nozzle temperature (°C)	Surface roughness SR^TB^ (µm)	Surface roughness SR^S^ (µm)	Printing time PT (min)
1	125.86	0.29	225.7	4.03101	1.73	23.20
2	126.42	0.29	224	3.94752	1.73	23.31
3	125.61	0.29	221	3.84666	1.72	23.47
4	125.83	0.29	226	4.00798	1.73	23.24
5	122.40	0.30	225.9	4.0432	1.72	23.22

### RSM solution

Using JMP software, the goal of output response was set to minimization with the importance of 0.25 of SR^TB^, 0.25 of SR^S^ and 0.5 of PT. The parameters range and levels are assigned to construct a CCD. The RSM predicts the parameter values to print a high surface quality component at a minimum printing time, as given in Table 5.

**Table 5:** RSM predicted results.

S. No	FFF parameters			Output responses		
	Printing speed (mm/s)	Layer thickness (mm)	Nozzle temperature (°C)	Surface roughness SR^TB^ (µm)	Surface roughness SR^S^ (µm)	Printing time PT (min)
1	76.98	0.25	220	3.23	1.51	32.15

### Evaluation of RSM and PSO results using WASPAS ranking method

Compared to the result of the Weighted Sum Model (WSM) and Weighted Product Model (WPM), the ranking accuracy was enhanced by combining both WSM and WPM. The efficiency of the RSM and PSO algorithms was evaluated by employing the WASPAS method. The optimum parameters predicted for the output responses (SR^TB^ of 25% weightage, SR^S^ of 25% weightage and PT of 50% weightage) by RSM and the PSO, which are given in Table 6. These values are compared using the WASPAS method to select the best algorithm and the optimum solution.

**Table 6:** RSM and PSO predicted results.

Optimization technique	FFF parameters			Output responses		
	Printing speed (mm/s)	Layer thickness (mm)	Nozzle temperature (°C)	Surface roughness SR^TB^ (µm)	Surface roughness SR^S^ (µm)	Printing time PT (min)
PSO	125.86	0.29	225.7	4.03101	1.73	23.20
	126.42	0.29	224	3.94752	1.73	23.31
	125.61	0.29	221	3.84666	1.72	23.47
	125.83	0.29	226	4.00798	1.73	23.24
	122.4	0.3	225.9	4.0432	1.72	23.22
RSM	76.98	0.25	220	3.23	1.51	32.15

The steps followed for the WASPAS method are shown below.

**Step 1:** Initialize of decision Matrix: The decision matrix *Y *is given in Eq. (13) where ‘*p*’ represents the No. of alternatives and ‘*q*’ represents the No. of criteria.

13 $$Y=\left[\begin{array}{ccc}{y}_{11}& {y}_{12} \dots & {y}_{1q}\\ \vdots & \vdots \vdots & \vdots \\ {y}_{p1}& {y}_{p2} \cdots & {y}_{pq}\end{array}\right]$$

To initialize the decision matrix, the top 5 optimum results were predicted by PSO and RSM algorithms, minimum SR^TB^, SR^S^ and PT values predicted by the mathematical model are taken to form a decision matrix as given in Table 7.

**Table 7:** WASPAS Method calculated Rank and alternatives scores.

S. No	Optimum result prediction method	FFF parameters			Output responses			${Q}_{i}^{(1)}$	${Q}_{i}^{(2)}$	${Q}_{i}$(λ = 0.5)	Rank
		Printing speed (mm/s)	Layer thickness (mm)	Nozzle temperature (°C)	Surface roughness SR^TB^ (µm)	Surface roughness SR^S^ (µm)	Printing time PT (min)				
1	**PSO**	125.86	0.29	225.7	4.03101	1.73	23.20	0.8437	0.8230	0.8334	5
2		126.42	0.29	224	3.94752	1.73	23.31	0.8444	0.8255	0.8349	2
3		**125.61**	**0.29**	**221**	**3.84666**	1.72	23.47	**0.8454**	**0.8286**	**0.8370**	**1**
4		125.83	0.29	226	4.00798	1.73	23.24	0.8438	0.8237	0.8337	3
5		122.4	0.3	225.9	4.0432	1.72	23.22	0.8439	0.8232	0.8336	4
6	**RSM**	79.6	0.25	220	3.24	31.60	1.52	0.7732	0.7691	0.7711	6

Significant values are in bold.

**Step 2:** Normalization of decision matrix using Eq. (14)

14 $${\mathrm{Minimization\,criteria }Y}_{ij}=\frac{min\,{y}_{ij}}{{y}_{ij}}$$

**Step 3:** Calculating (${Q}_{i}^{(1)}$) the total relative importance through WSM using Eq. (15)

15 $${Q}_{i}^{(1)}= \sum_{j=1}^{n}{y}_{ij} . {w}_{j}$$

**Step 4:** Calculating (${Q}_{i}^{(2)}$) the total relative importance through WPM using Eq. (16)

16 $${Q}_{i}^{(2)}= \sum_{j=1}^{n}{{(y}_{ij})}^{{w}_{j}}$$

**Step 5:** Calculating the total relative significance of alternatives using Eq. (17)

17 $${Q}_{i}= \lambda . {Q}_{i}^{(1)}+\left(1-\lambda \right) .{Q}_{i}^{\left(2\right)}$$

The highest value of *Qi* represents the best alternative^44^. The WASPAS ranking method given in Table 7 shows that the 3rd alternative got the highest Rank − 1 when compared with other alternatives. Hence 3rd alternative predicted by PSO was taken as an optimum solution to enhance the surface quality of 3D printed components at minimum printing time. The WASPAS result shows that the PSO algorithm predicted result got the top 5 ranks, whereas the RSM predicted result got the 6th rank. From this, it was clearly understood that PSO is an effective algorithm to solve multi-objective functions when compared to RSM.

### Evaluation of PSO results with the experiment results

To evaluate the PSO predicted result a specimen was fabricated for the printing speed of 125.6 mm/s, the layer thickness of 0.29 mm and the nozzle temperature of 221 °C. The measured average Surface Roughness SR^TB^ (µm), average Surface Roughness SR^S^ (µm) and Printing Time PT (min) were depicted in Table 8, and compared the PSO predicted results with the experimental results. The error % between the PSO predicted results and experimental results were also calculated, which are less than 4%.

**Table 8:** PSO results versus experiment results.

S. No	FFF parameters			Output responses			WASPAS rank
	Printing speed (mm/s)	Layer thickness (mm)	Nozzle temperature (°C)	Avg. surface roughness SR^TB^ (µm)	Avg. surface roughness SR^S^ (µm)	Printing time PT (min)	
1	125.6	0.29	221	**PSO predicted result**			1
				3.84	1.72	23.47	
				**Experimental result**			
				3.92	1.78	24	
	**Error %**			2	3.4	2.2	

The error % less than 4 indicates that the developed model can predict the optimal solution. Moreover, compared to RSM, applying the PSO algorithm is an effective approach to solve the multi-objective problem.

### Effect of process parameters on output responses

The objective of this research is to predict optimum FFF process parameters to enhance the FFF component surface quality at minimum printing time. The effect of process parameters, such as printing speed (mm/s), layer thickness (mm) and nozzle temperature (°C) on output responses (Surface Roughness SR^*TB*^, Surface Roughness SR^*S*^ and Printing Time (min)) was analyzed using the ANOVA statistical analysis tool.

### Process parameters versus surface roughness SR^TB^

The ‘F’ (91.81) value of the model developed for SR^TB^ derived through the ANOVA tool, and the R^2^ value of 96% implies that the model is reliable. Moreover, the ‘*p*’ value of all three process parameters was less than 0.0001 which indicates that all three parameters influence the SR^TB^. Compared to the printing speed ‘F’ (39.1) value and the nozzle temperature ‘F’ (74.4) value, the ‘F’ (161.9) value of layer thickness was higher which indicates that the parameter layer thickness has a high influence on SR^TB^. Moreover, compared to high printing speed (160 mm/s), the specimen printed at low speed (60 mm/s) exhibits high surface quality. On the one hand, higher printing speed with low extruder temperature induces under extrusion which increases the surface roughness. Similarly, the high printing speed with high extruder temperature causes over extrusion which produced a stringing effect with poor surface quality. Higher printing speed also induced vibration while printing which causes dimensional inaccuracies and poor surface quality. Compared to high nozzle temperature (245 °C), the specimen printed at low nozzle temperature (220 °C) exhibits high surface quality. At high nozzle temperature over extrusion take place which increases the surface roughness. Also, the surface roughness value was highly influenced by varying layer thickness. The surface roughness values were minimal on top and bottom surfaces compared to side surfaces due to stair casing effect. The specimen fabricated with a minimum layer thickness (0.1 mm) exhibited high surface quality, compared with a specimen fabricated with a maximum layer thickness (0.3 mm). Due to over extrusion at maximum layer thickness, the surface quality was reduced on FFF printed components.

### Process parameters versus surface roughness SR^S^

The ‘F’ (313.42) value of the model developed for SR^S^ through the ANOVA tool, and the R^2^ value of 99% implies that the model is reliable. Moreover, the ‘*p*’ value of printing speed and layer thickness were less than 0.0001 which indicate that these parameters highly influence the SR^S^. The ‘*p*’ value of nozzle temperature was 0.128  that indicates it is not significant to response SR^S^. Compared to layer thickness ‘F’ (75.67) value, the ‘F’ (161.9) value of printing speed was high, which indicates the parameter printing speed has high influence on the SR^S^. Compared to high printing speed (160 mm/s), the specimen print at low speed (60 mm/s) exhibited better surface quality. At higher printing speed under extrusion take place which increased the surface roughness and bonding strength between layers. The specimen fabricated with a minimum layer thickness (0.1 mm) exhibits better surface quality, compared with the specimen fabricated with a maximum layer thickness (0.3 mm).

### Process parameters versus printing time PT

The ‘F’ (212.62) value of the model developed for PT derived through ANOVA, and the R^2^ value of 100% implies that the model is significant. Moreover, the ‘*p*’ value printing speed and layer thickness were less than 0.0001 which indicates that these parameters highly influence the PT. The ‘*p*’ value of nozzle temperature is 1.00 that indicates it is not significant to response PT. Compared to the printing speed ‘F’ (11.14) value, the ‘F’ (29.7) value of layer thickness was higher which indicates the parameter layer thickness has high influence on PT. The specimen fabricated with maximum print speed and minimum layer thickness reduces the print time drastically. The specimen fabricated with maximum print speed and maximum layer thickness exhibit minimum printing time.

## Conclusions

In this research, multi-optimization techniques were applied to predict the optimum FFF parameters in improving the surface quality of the flat upper and down, and side surfaces of FFF components at minimum printing time. Two important methods, such as RSM and PSO methods were employed in this research, to predict the optimum printing parameters using the ANOVA statistical analysis tool. The weightage of 0.25, 0.25 and 0.5 was assigned for the output responses SR^TB^, SR^S^ and PT using the Equal-weights method. For the given weightage, the RSM predicts the printing speed of 76.98 mm/sec, nozzle temperature of 220 °C and layer thickness of 0.25 mm and the corresponding output response was SR^TB^ of 3.23 µm, SR^S^ of 1.51 µm and PT of 32.15 min. For the given weightage, the PSO predicts the printing speed of 125.6 mm/sec, nozzle temperature of 221 °C and layer thickness of 0.29 mm and the corresponding output response was SR^TB^ of 3.84 µm, SR^S^ of 1.72 µm and PT of 23.47 min. The WASPAS ranking method was employed to compare the RSM and PSO to identify the best output responses. Moreover, through the WASPAS ranking method, it was understood that compared to RSM, the PSO algorithm is more effective to solve multi-objective problems. The predicted results were compared with the experimental results, and obtained good correlations with the percentage of deviation of less than 4%. The results presented in this study will be useful to develop several optimization-based statistical models for minimizing the number of experimental trials by controlling the additive manufacturing process parameters.

## Supplementary Information

Supplementary Information 1.

Supplementary Information 2.

## Data Availability

All data generated or analyzed during this study are included in this article [and its supplementary information files.
